# Supplementary material for: Pantothenate kinase 4 controls skeletal muscle substrate metabolism
Source: Nat Commun. 2025 Jan 2;16:345. doi: 10.1038/s41467-024-55036-w (PMC11695632; doi:10.1038/s41467-024-55036-w)
Supplement: Supplementary file 1 — SUPPLEMENTARY INFO [file 41467_2024_55036_MOESM1_ESM.pdf]

Supplementary Information

Pantothenate kinase 4 controls skeletal muscle substrate metabolism

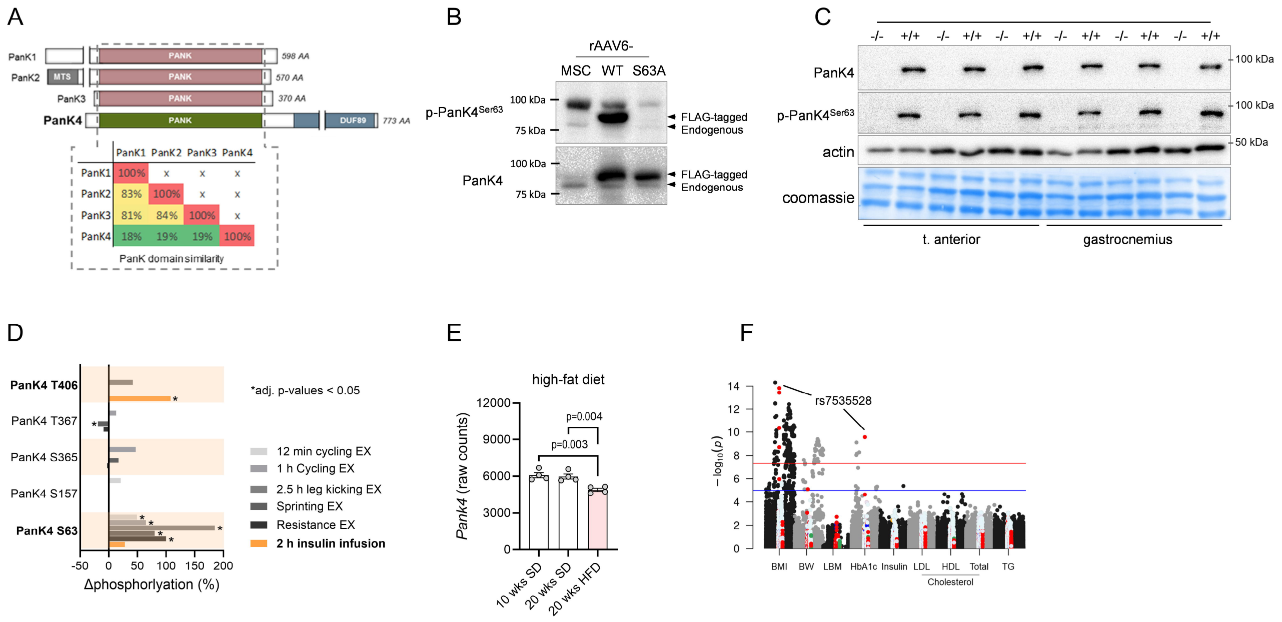

Supplementary Fig. 1. **a,** schematic of pantothenate kinase (PanK) proteins and the %PanK domain similarity at the amino acid level among PanK1-4. **b,** total PanK and PanK4 phosphorylation at Ser63 (p-PanK4^{Ser63}) in tibialis anterior muscles from male C57BL/6J mice that were i.m. injected with recombinant adeno-associated virus serotype 6 (rAAV6) encoding either wildtype PanK4 (rAAV6:WT), a mutant PanK4 in which Ser63 was exchanged with alanine (rAAV6:S63A), or rAAV6:MCS as control. **c,** total PanK and p-PanK4^{Ser63} in indicated muscles from PanK4 WT or PanK4 KO mice. **d**, phosphorylation levels determined by phosphoproteomics of indicated sites on PanK4 and their regulation by exercise or insulin infusion in skeletal muscle from humans[13,37,52]. **e**, *Pank4* in skeletal muscle from mice fed a standard diet (SD) or a high-fat diet for indicated number of weeks (n = 4). **f,** single-nucleotide polymorphisms (SNPs) 50kb up- and downstream of the human *PANK4* gene and their associations with indicated traits. Red circles are SNPs within *PANK4*. Red horizontal line indicates threshold for genome wide association. Statistic: e, one-way ANOVA with log2-transformed data and Šidák post hoc test; data are presented as mean values +/-SEM. Source data are provided as a Source Data file.

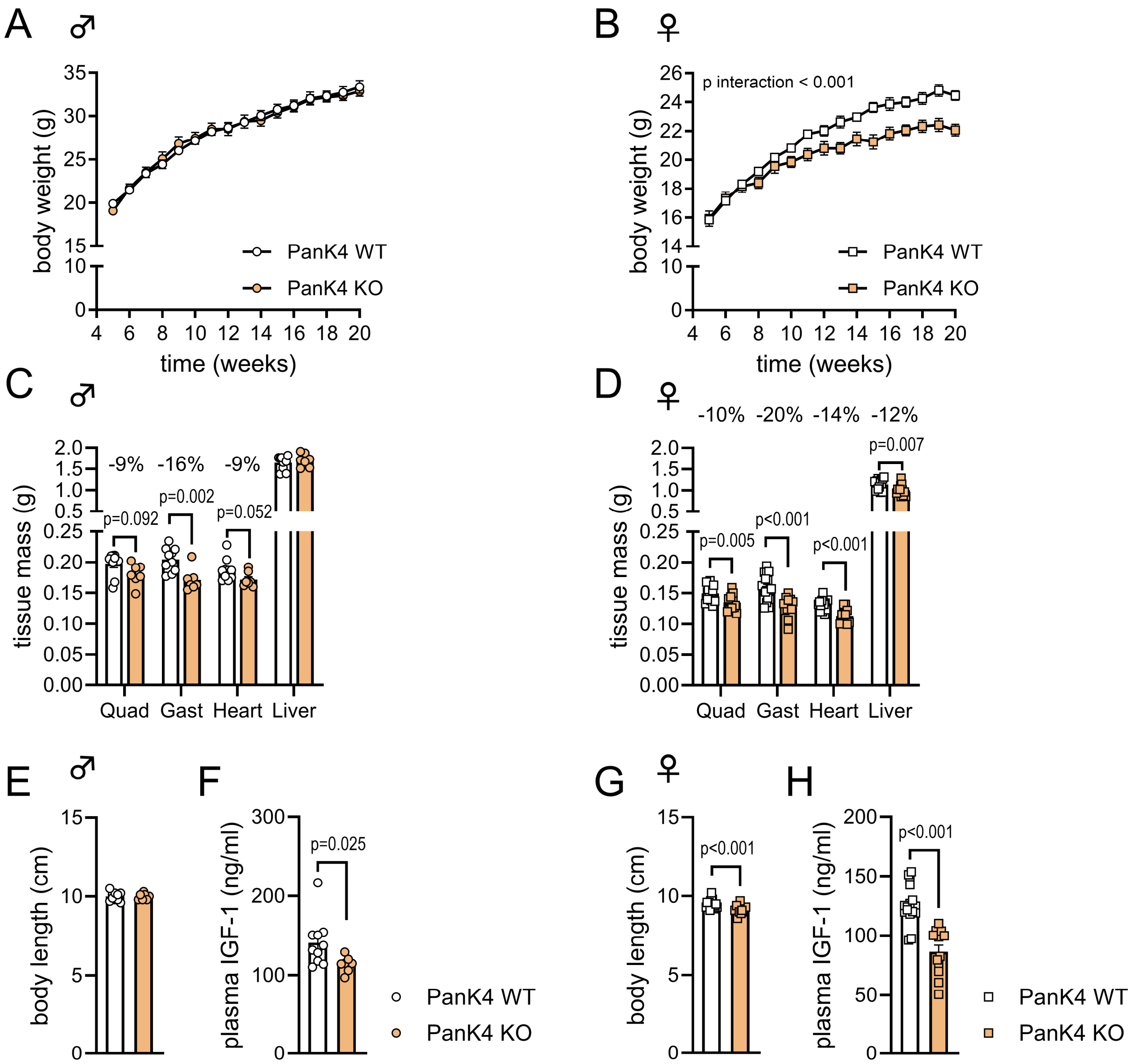

Supplementary Fig. 2. a,b, body weight over time in chow-fed male (n = 11 for WT and n = 7 for KO) or female (n = 16 for WT and n = 12 for KO) PanK4 WT and PanK4 KO mice. **c,d,** mass of indicated tissues in fed PanK4 WT and KO mice at age 24 weeks (male: n = 10 for WT and n = 7 for KO; female: n = 16 for WT and n = 12 for KO). **e-h** body length and plasma IGF-1 concentration in fed PanK4 WT and KO mice at age 24 weeks (male: n = 10 for WT and n = 7 for KO; female: n = 16 for WT and n = 12 for KO). Statistic: a,b, repeated measures two-way (time x genotype) ANOVA; c,d, two-tailed unpaired t-tests within tissue; e-h, two-tailed unpaired t-test. Statistical analyses were conducted with log2-transformed data. Data are presented as mean values +/-SEM. Source data are provided as a Source Data file.

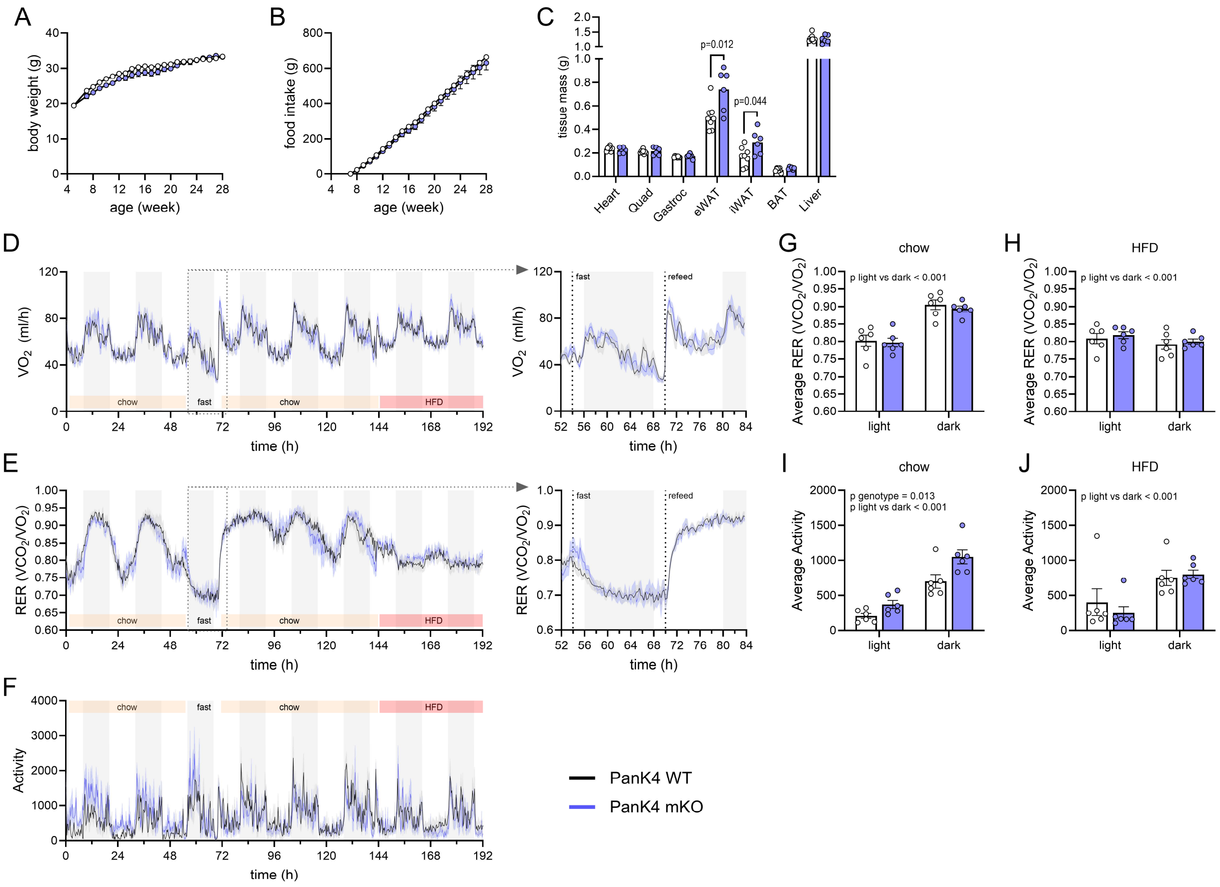

Supplementary Fig. 3. **a,b**, body weight and food intake of male chow-fed PanK4 WT and mKO mice over time (n = 8 for WT and n = 6 for mKO). **c**, wet weights of indicated tissues from PanK4 WT and PanK4 mKO mice at age 28 weeks (n = 8 for WT and n = 6 for mKO). **d-f**, Oxygen consumption (VO_2), respiratory exchange ratio (RER), locomotor activity during indicated feeding regimes (gray shading indicated dark phase, n = 6). **g-j**, average RER and average activity during light and dark phases and indicated diet regimes (n = 6). Statistic: c, two-tailed unpaired t-test within organ/tissue. g-j, repeated measure two-way (genotype x phase) ANOVA. Statistical analyses were conducted with log2-transformed data. Data are presented as mean values +/-SEM. Source data are provided as a Source Data file.

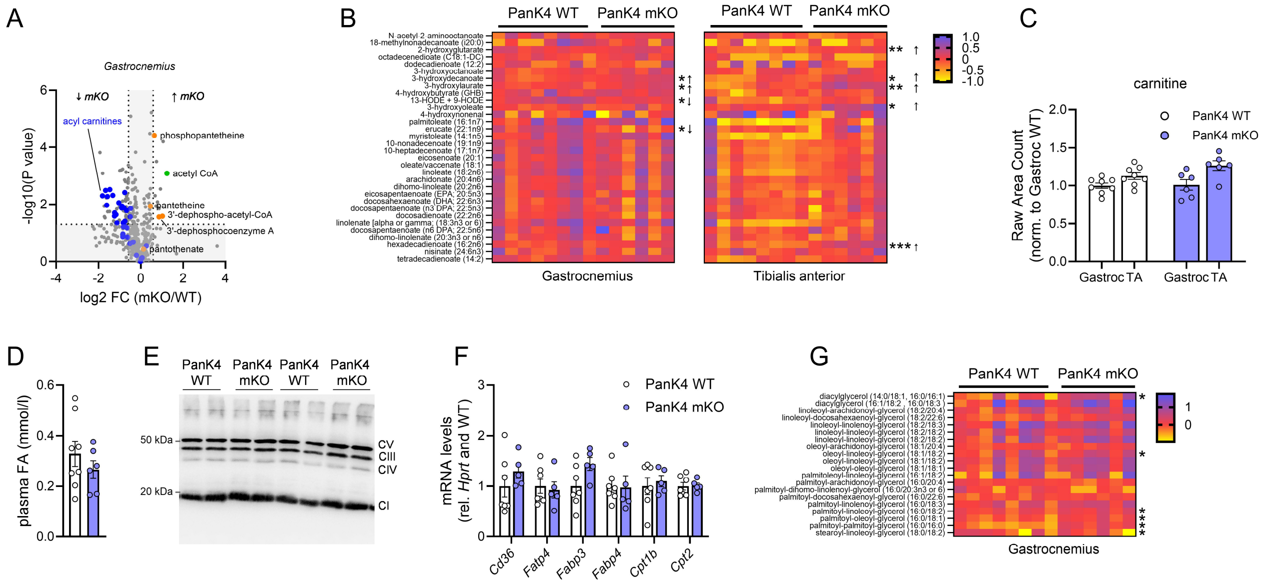

Supplementary Fig. 4. a,b, volcano plot (a) of 508 detected metabolites in gastrocnemius muscles and heatmap (b) of indicated metabolites in indicated muscles from male mcie (n = 8 for WT and n = 6 for mKO). **c,** carnitine determined by non-targeted metabolomics in male PanK4 WT and PanK4 mKO mice at age 28 weeks (n = 8 for WT and n = 6 for mKO). **d,** plasma fatty acids (FA) from male PanK4 WT and PanK4 mKO mice at age 28 weeks (n = 8 for WT and n = 6 for mKO). **e,** OXPHOS western blot in gastrocnemius muscles from male glucose-stimulated PanK4 WT and PanK4 mKO mice at age 28 weeks. **f**, mRNA abundance of indicated genes in gastrocnemius muscles from male glucose-stimulated PanK4 WT and PanK4 mKO mice at age 28 weeks (n = 7 for WT and n = 5 for mKO). **g,** heatmap of indicated metabolites in indicated muscle (n = 8 for WT and n = 6 for mKO) from male glucose-stimulated PanK4 WT and PanK4 mKO mice at age 28 weeks. ***p<0.001, **p<0.01, *p<0.05 vs corresponding PanK4 WT. Statistic: a,b,g, two-tailed welch test; c-f, data are presented as mean values +/-SEM. Source Data are provided as a Source Data file.

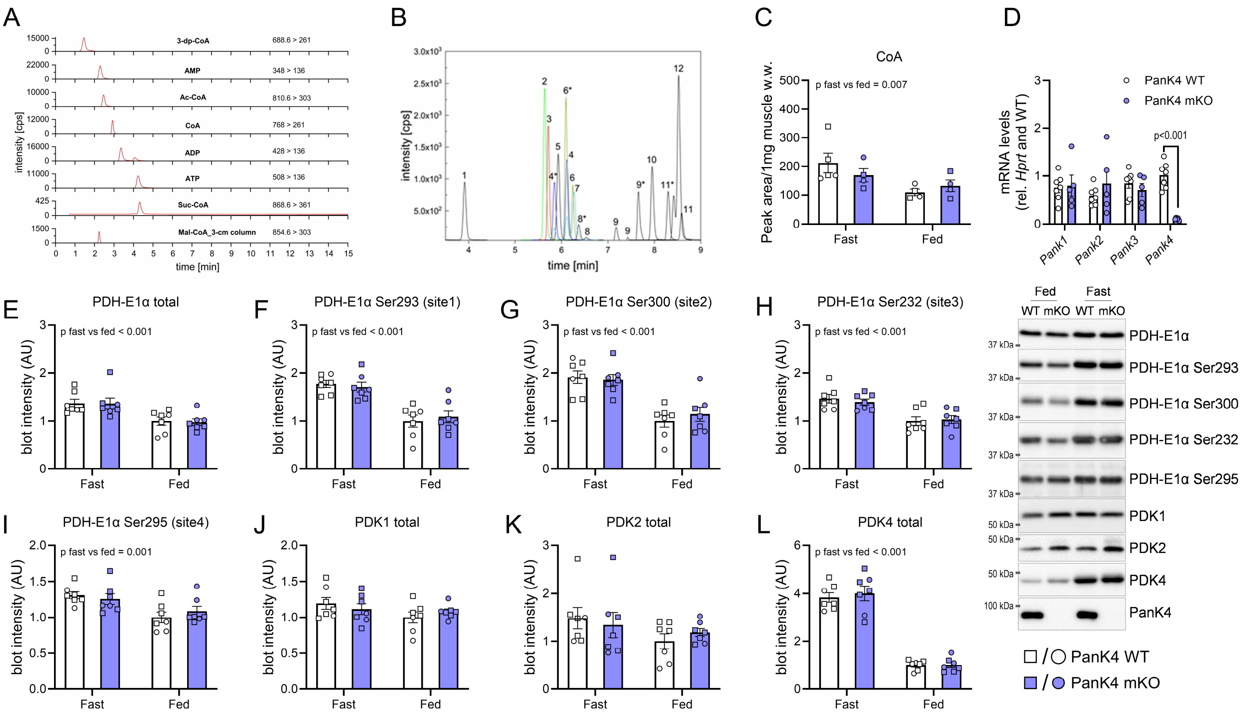

Supplementary Fig. 5. **a,** MRM chromatograms of a 1 µM standard mixture of phosphate-containing metabolites analyzed on an iHILIC-(P) Classic column, 50 × 2.1 mm, 5 µm; **b,** dynamic MRM chromatogram of a 100 nM standard mixture of 3-NPH-derivatized carboxylic acids analyzed on an HSS T3 column. Peak assignment: 1 – lactic acid, 2 – malic acid, 3 – 2-hydroxyglutaric acid, 4 – isocitric acid, 5 – succinic acid, 6 – itaconic acid, 7 – citric acid, 8 – fumaric acid, 9 – oxaloacetic acid, 10 – 2-oxoglutaric acid. Instrumental conditions as described in LC-MS conditions. Asterisk denotes an integration peak used for quantification of carboxylates represented by multiple peaks in chromatogram. **c,** targeted analysis of Coenzyme A (CoA) in gastrocnemius from fasted and fed male (circles) and female (squares) PanK4 WT and PanK4 mKO mice (n = 5 for WT/fast, n = 4 for WT/fed, n = 4 for mKO/fast, n = 4 for mKO/fed). **d,** mRNA abundance of indicated genes in gastrocnemius muscles of PanK4 WT and PanK4 mKO mice (n = 7 for WT and n = 5 for mKO). **e-l**, quantification of western blot results and representative blots for indicated proteins or phosphorylation sites in gastrocnemius muscles from fasted or fed PanK4 WT and PanK4 mKO mice (n = 7); male (circles) and female (squares) mice were used. Statistic: c, e-l, two-way (fast/fed x genotype) ANOVA with log2-transformed data. Data are presented as mean values +/-SEM. Source data are provided as a Source Data file. AU = arbitrary units.

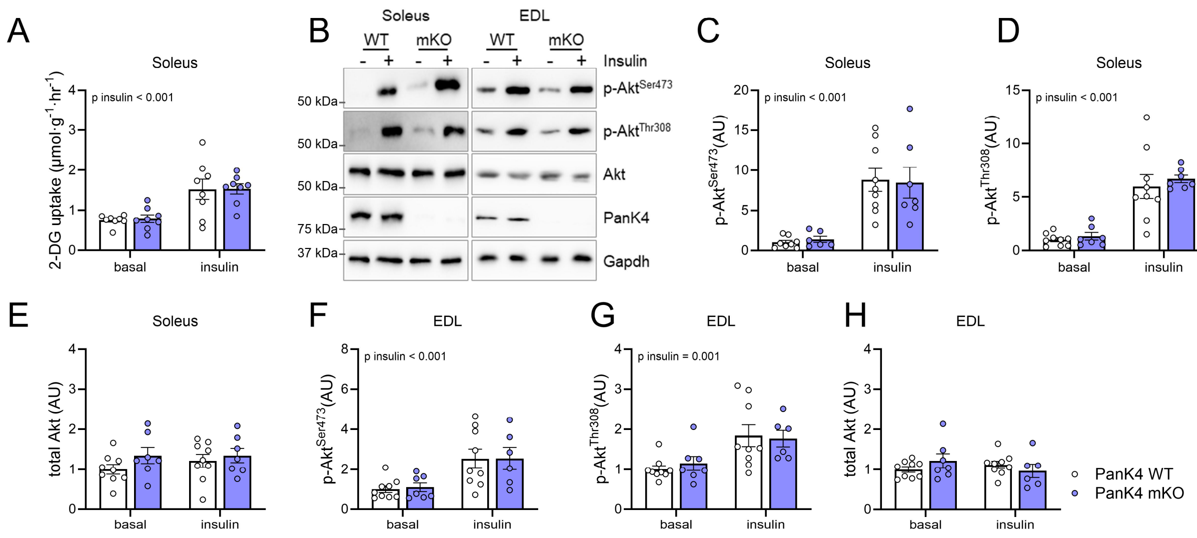

Supplementary Fig. 6. **a,** insulin-stimulated glucose uptake in soleus from male PanK4 WT and PanK4 mKO incubated ex vivo under basal conditions or stimulated with 1.8 nM insulin (n = 8). **b-h,** representative western blots and quantification relative to baseline WT of indicated phosphorylation sites or protein in insulin-stimulated soleus and EDL muscles; c (n = 8 for WT/basal, n = 9 for WT/insulin, n = 6 for mKO/basal, n = 7 for mKO/insulin); d (n = 9 for WT/basal, n = 9 for WT/insulin, n = 7 for mKO/basal, n = 7 for mKO/insulin); e (n = 9 for WT/basal, n = 9 for WT/insulin, n = 7 for mKO/basal, n = 7 for mKO/insulin); f (n = 9 for WT/basal, n = 9 for WT/insulin, n = 7 for mKO/basal, n = 6 for mKO/insulin); g (n = 9 for WT/basal, n = 9 for WT/insulin, n = 7 for mKO/basal, n = 6 for mKO/insulin), h (n = 9 for WT/basal, n = 9 for WT/insulin, n = 7 for mKO/basal, n = 6 for mKO/insulin). Statistic: a, c-h, two-way (genotype x insulin) ANOVA with log2-transformed data. Data are presented as mean values +/-SEM. Source data are provided as a Source Data file. AU = arbitrary units.

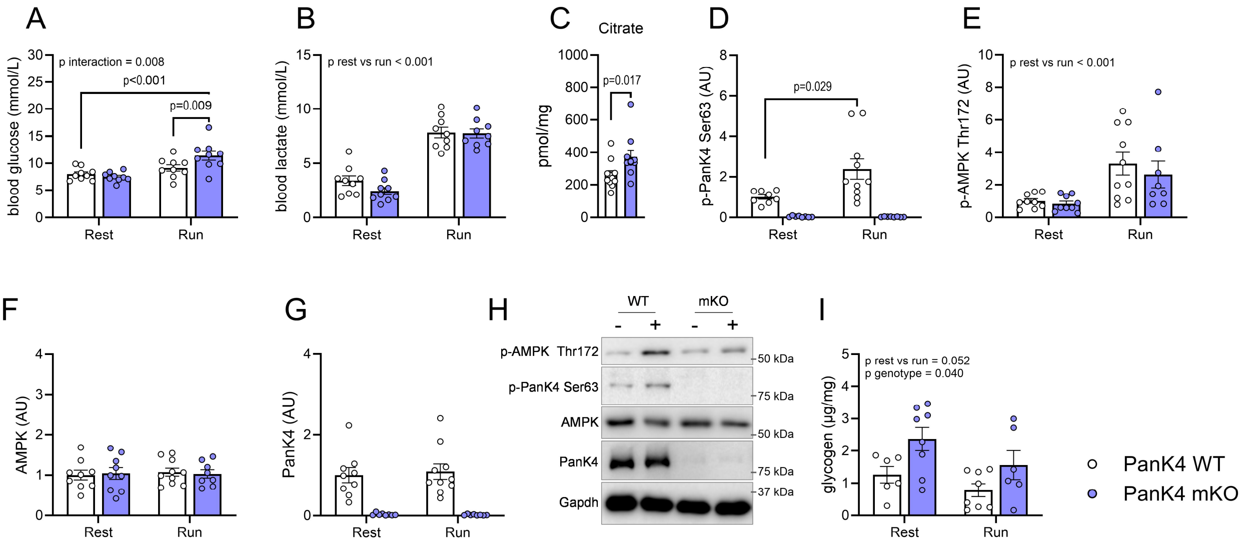

Supplementary Fig. 7. a,**b**, blood glucose (a) and blood lactate (b) determined before and right after a maximal running capacity test in male PanK4 WT and PanK4 mKO mice (n = 9). **c**, citrate levels determined by targeted analyses in quadriceps muscle from male PanK4 WT and PanK4 mKO mice that had ran on the treadmill (n = 11 for WT and n = 10 for mKO). **d-i**, quantification of western blots and representative blots of indicated proteins or phosphorylation sites (d-h), d (n = 8 for WT/rest, n = 10 for WT/run, n = 9 for mKO/rest, n = 8 for mKO/run), e (n = 9 for WT/rest, n = 10 for WT/run, n = 9 for mKO/rest, n = 8 for mKO/run), f (n = 9 for WT/rest, n = 10 for WT/run, n = 9 for mKO/rest, n = 8 for mKO/run), g (n = 9 for WT/rest, n = 10 for WT/run, n = 9 for mKO/rest, n = 8 for mKO/run); and glycogen (i, n = 6 for WT/rest, n = 8 for WT/run, n = 8 for mKO/rest, n = 6 for mKO/run)) in quadriceps muscles from male PanK4 WT and PanK4 mKO mice after 20 min of rest or treadmill running at 75% of maximal running capacity. Statistic: a,b, e, i, two-way (genotype x activity) ANOVA with log2-transformed data and Šidák post hoc testing; c, two-tailed student's t-test with log2-transformed data; d, two-tailed student's t-test comparing rest vs. run with PanK4 WT with log2-transformed data. Data are presented as mean values +/-SEM. Source data are provided as a Source Data file. AU = arbitrary units.

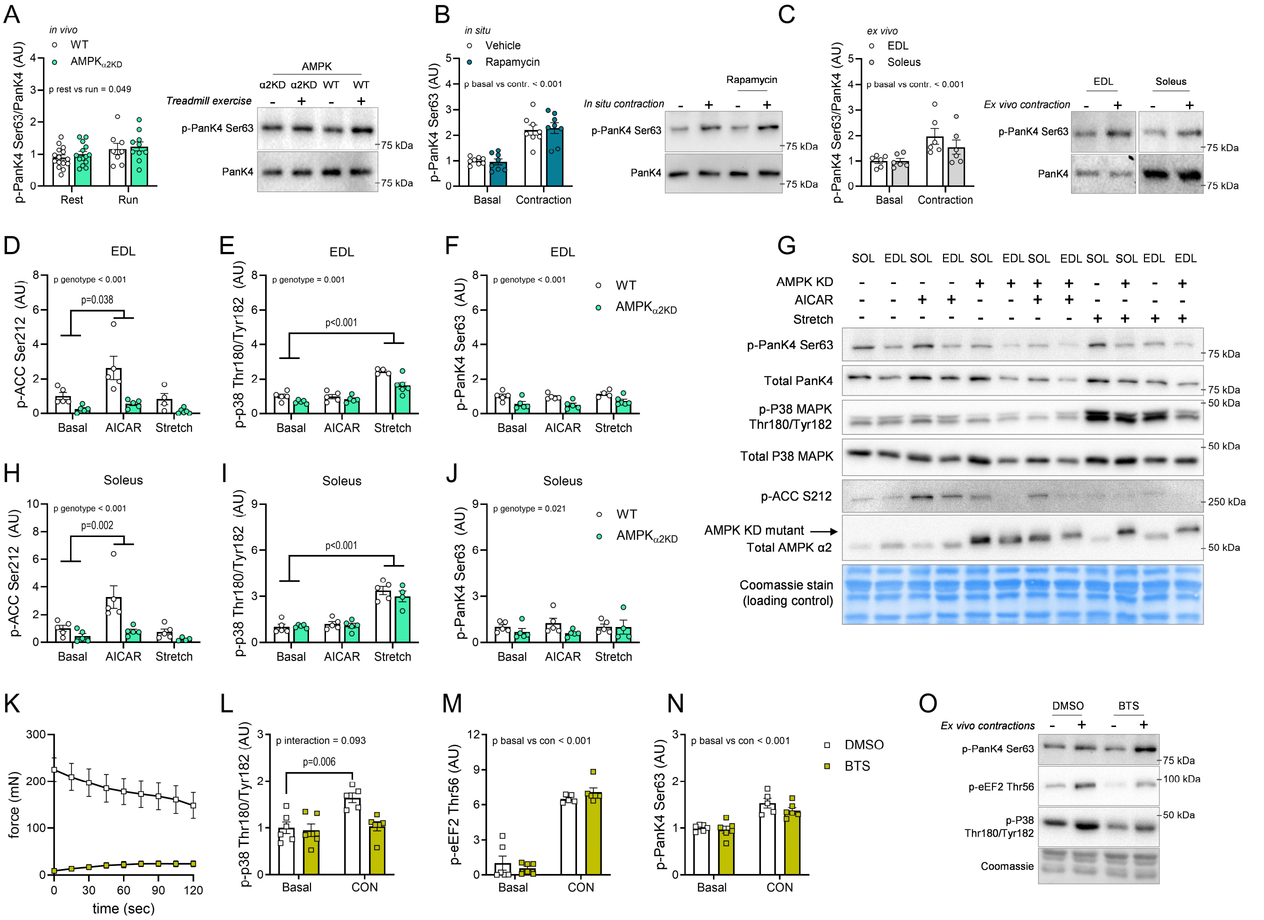

Supplementary Fig. 8. a-c, effect of treadmill running (a, n = 15 WT/rest, n = 8 WT/run, n = 14 KD/rest, n = 10 KD/run), *in situ* (b, n = 8), and *ex vivo* (c, n = 6) contractions on muscle p-PanK4^{Ser63} with quantification and representative blots of indicated proteins. **d-j**, effect of basal, AICAR, and stretch on p-PanK4^{Ser63} and indicated proteins and phosphorylation sites in EDL (d-e, n = 5 for WT/basal, n = 5 for WT/AICAR, n = 4 for WT/stretch, n = 5 for KD/basal, n = 5 for KD/AICAR, n = 6 for KD/stretch) and soleus (h-j, n = 5 for WT/basal, n = 5 for WT/AICAR, n = 5 for WT/stretch, n = 5 for KD/basal, n = 5 for KD/AICAR, n = 4 for KD/stretch) muscles from wildtype or muscle-specific transgenic mice overexpressing a kinase-dead α2 subunit of AMPK (AMPK KD) incubated *ex vivo*. **k-n**, effect of electrically-stimulated *ex vivo* contractions or blockade of force development by blebbistatin (BTS) on force production (k, n = 4) and phosphorylation of indicated sites (l-n, n = 6 for DMSO/basal, n = 5 for DMSO/con, n = 6 for BTS/basal, n = 6 for BTS/con) in EDL muscles. Statistic: a, two-way (genotype x activity) ANOVA; b, two-way (genotype x contraction) ANOVA; c, two-way (muscle x contraction) ANOVA; d-j, two-way (genotype x basal/AICAR/stretch) ANOVA; k-n, two-way (inhibitor x contraction) ANOVA with log2-transformed data and Šidák post hoc testing. Data are presented as mean values +/-SEM. Source data are provided as a Source Data file. AU = arbitrary units.

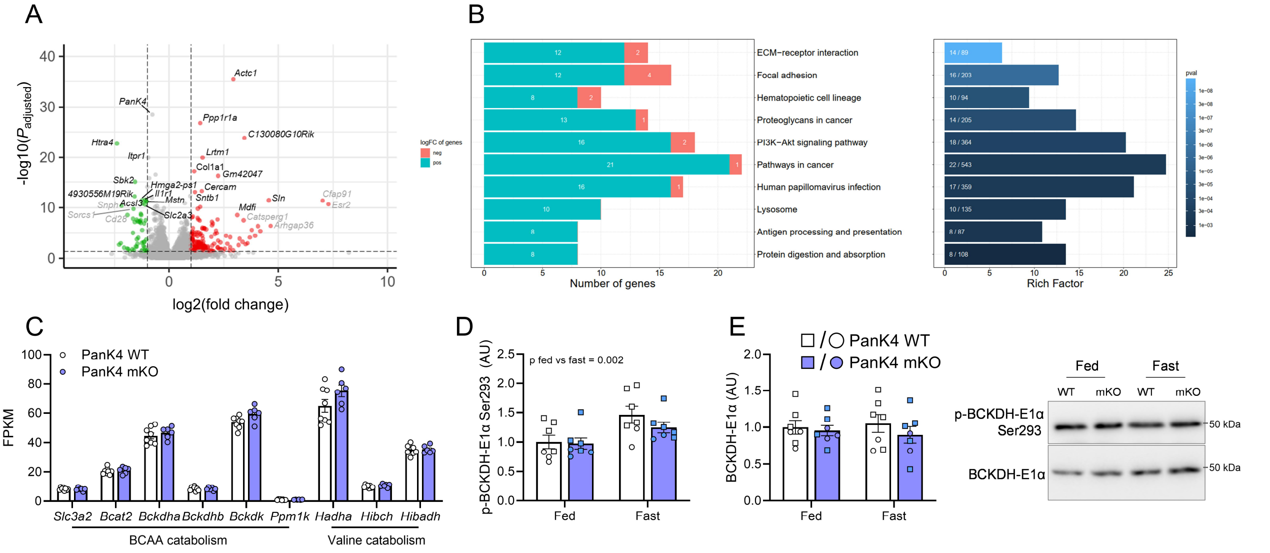

Supplementary Fig. 9. a-b, whole-genome RNA-sequencing of gastrocnemius muscle from male glucose-stimulated PanK4 WT and PanK4 mKO mice at age 28 weeks with a volcano plot (a) depicting detected genes over the threshold of 1 FPKM and the top ten most significantly enriched metabolic KEGG pathways (b) (n = 6-8). **c**, expression of indicated genes in PanK4 WT and PanK4 mKO SkM based on the whole-genome RNA-sequencing data (n = 8 for WT, n = 6 for mKO). **d,e**, representative western blots and quantification of indicated phosphorylation site and protein in PanK4 WT and PanK4 mKO muscle from fed or fasted male (circles) and female (squares) mice (n = 7). Statistic: a, differentially gene expression was calculated using DESeq2 (V1.34.0); b, pathway enrichment was performed with the DAVID tool; d,e, two-way (genotype x prandial status) ANOVA with log2-transformed data. c-e, data are presented as mean values +/-SEM. Source data are provided as a Source Data file. AU = arbitrary units.

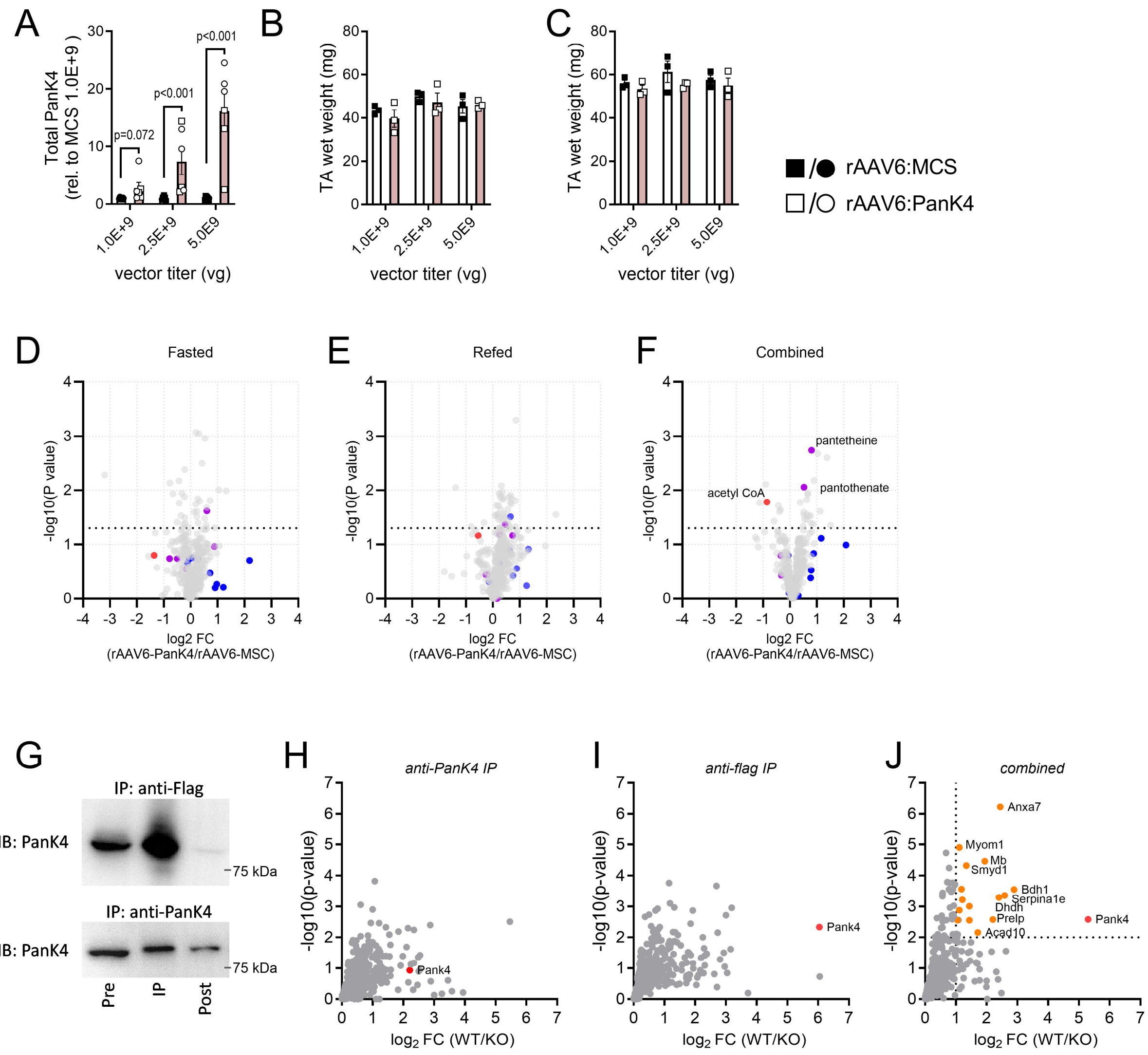

Supplementary Fig. 10. a-c, dose-response study looking at the effect of AAV vector titer on PanK4 abundance in TA muscle: male (circles) and female (squares) C57BL/6J mice were injected into TA with indicated titers of recombinant adeno-associated viral vector serotype 6 encoding PanK4 (rAAV6:PanK4), while contralateral TA was injected with rAAV6:MCS as control. Mice were killed 14 days later and (a) PanK4 abundance was assessed by western blot (n = 6) and (b,c) TA mass was determined (n = 3). **d-f**, volcano plots of metabolites detected by metabolomics in TA muscles from fasted and refed male C57BL/6J mice with rAAV6:PanK4 in one and rAAV6:MCS in contralateral TA (1.0E+9 vg) (n = 6). **g**, representative western blot of PanK4 after immunoprecipitation (IP) in lysates from rAAV6:PanK4 TA muscles with anti-Flag or PanK4 antibody. **h-j**, one-side volcano plots of proteins detected in the IP samples (g) by mass spectrometry compared to IP performed in TA from PanK4 KO mice. Statistic: a-c, two-way (titer x rAAV6) ANOVA with log2-transformed data and Šidák post hoc testing; d-f,h,i, two-tailed welch test. Source data are provided as a Source Data file.

Supplementary Table 1

Gene	Forward sequence (5´- 3´)	Reverse Sequence (5´- 3´)
Pank4	GAGACCACCTAGTCAACACTGA	GCAGGTCATTACATCCTCTTTGT
Pank1	GTTCGCCCAGCATGATTCTC	CTTAACCAGGGTTCCACCGAT
Pank2	GAGGCGGAGAGTGTGAGAC	CCAAGGTTCCTCCAATATCCAAG
Pank3	GGACATTGGAGGAACGCTAGT	ACATCCCGAATGCCAGTAGAT
Hprt	TCAGTCAACGGGGGACATAAA	GGGGCTGTACTGCTTAACCAG
Gdf15	CCGAGAGGACTCGAACTCAG	GGTTGACGCGGAGTAGCAG
Cd36	ATGGGCTGTGATCGGAACTG	GTCTTCCCAATAAGCATGTCTCC
Fapb3	ACCTGGAAGCTAGTGGACAG	TGATGGTAGTAGGCTTGGTCAT
Fabp4	TGAAATCACCGCAGACGACA	ACACATTCCACCACCAGCTT
Fatp4 (Slc27a4)	ACTGTTCTCCAAGCTAGTGCT	GATGAAGACCCGGATGAAACG
Cpt1b	GCACACCAGGCAGTAGCTTT	CAGGAGTTGATTCCAGACAGGTA
Cpt2	CAGCACAGCATCGTACCCA	TCCCAATGCCGTTCTCAAAAT
Fgf21	GCTGCTGGAGGACGGTTACA	CACAGGTCCCCAGGATGTTG

Supplementary Table 2

Antibody	
PanK4	PANK4 (D8N2C) Rabbit mAb #12055; Cell Signaling
Beta-actin	β-Actin Antikörper (C4) HRP sc-47778; Santa Cruz Biotechnolgy
p-PanK4 Ser63	Custom (Maximilian Kleinert)
pan-lysine acetylation	Acetylated-Lysine Mouse mAb (Ac-K-103) #9681s; Cell Signaling
pan-lysine malonylation	Malonyl-Lysine [Mal-K] MultiMab Rabbit mAb mix #14942; Cell Signaling
p-Akt Ser473	Phospho-Akt (Ser473) (D9E) XP® Rabbit mAb #4060; Cell Signaling
p-Akt Thr308	Phospho-Akt (Thr308) (244F9) Rabbit mAb #4056; Cell Signaling
Akt	Akt Antibody #9272; Cell Signaling
Oxphos	Total OXPHOS Rodent WB Antibody Cocktail (ab110413); Abcam
GAPDH	GAPDH (G-9) HRP Mouse mAb # sc-365062; Santa Cruz Biotechnology
GLUT4	GLUT4 Polyclonal Antibody #PA-23052; ThermoFisher Scientific
Hexokinase II	Hexokinase II (C64G5) Rabbit mAb #2867; Cell Signaling
PDH-E1	Custom (G. Hardie, University of Dundee, Scotland)
PDH-E1 Ser293	Custom (G. Hardie, University of Dundee, Scotland)
PDH-E1 Ser300	Custom (G. Hardie, University of Dundee, Scotland)
PDH-E1 Ser295	Custom (G. Hardie, University of Dundee, Scotland)
PDK4	Custom (G. Hardie, University of Dundee, Scotland)
PDH-E1 Ser232	CalBioChem (acquired by Sigma, Sigma acquired by MERCK, Darmstadt, Germany) (AP1063)
PDK1	Abcam, Oxford, UK. (ab90444)
PDK2	CalBioChem (acquired by Sigma, Sigma acquired by MERCK, Darmstadt, Germany) (S1643)
BCKDH-E1α	BCKDH-E1α (E4T3D) Rabbit mAb #90198; Cell Signaling
BCKDH-E1α Ser293	Phospho-BCKDH-E1α (Ser293) (E2V6B) Rabbit mAb #40368; Cell Signaling

Supplementary Table 3 Multiple reaction monitoring parameters and retention times of phosphate-containing metabolites

Analyte	Retention time [min]	Precursor ion	Product ion	Collision energy [V]	Dwell time [ms]	Type of transition
**3-dp-CoA**	1.46	688.6	261	25	25	quantifier
			348	21	25	qualifier
**AMP**	2.29	348	136	17	25	quantifier
			97	41	25	qualifier
**Ac-CoA**	2.45	810.6	303	33	25	quantifier
			428	25	25	qualifier
**CoA**	2.95	768	261	33	25	quantifier
			428	25	25	qualifier
**ADP**	3.36	428	136	29	25	quantifier
			348	17	25	qualifier
**ATP**	4.29	508	136	39	25	quantifier
			410	17	25	qualifier
**Suc-CoA**	4.32	868.6	361	41	25	quantifier
			428	29	25	qualifier
**Mal-CoA***	2.70	854.6	303	42	25	quantifier
			347.2	34	25	qualifier
**AMP-$^{13}C_{10}$ $^{15}N_5$**	2.29	363	146	17	25	quantifier
**Ac-CoA-$^{13}C_2$**	2.45	812.5	305.2	38	25	quantifier
**ADP-$^{15}N_5$**	3.36	433	141	29	25	quantifier
**ATP-13C10 15N5**	4.29	523	146	39	25	quantifier
**Mal-CoA-$^{13}C_3$**	2.70	857.6	350	42	25	quantifier

* quantified on a shorter, 3-cm column

Supplementary Table 4 Multiple reaction monitoring parameters and retention times of 3-NPH-derivatized carboxylic acids

Analyte	Precursor	Products	Collision energy [V]	Type of transition	Retention time [min]
**oxaloacetic acid**	536	247.2	25	Quantifier	8.30
		357.1	25	qualifier	
**malic acid**	403	208.1	17	Quantifier	5.64
		180.0	29	qualifier	
**citric acid**	443	246.1	21	Quantifier	6.28
		177.8	17	qualifier	
**isocitric acid**	443	207.9	9	Quantifier	5.86
		136.9	49	qualifier	

**pyruvic acid**	357	42.0<br>136.8	29<br>29	Quantifier<br>qualifier	7.95
**2-oxoglutaric acid**	550	233.1<br>137.2	30<br>30	Quantifier<br>qualifier	8.53
**fumaric acid**	385	232.0<br>95.9	17<br>29	Quantifier<br>qualifier	6.38
***cis*-aconitic acid**	578	383.1<br>427.0	21<br>21	Quantifier<br>qualifier	7.63
**succinic acid**	387	234.0<br>98.0	21<br>33	Quantifier<br>qualifier	5.93
**lactic acid**	224	152.0<br>137.0	13<br>21	Quantifier<br>qualifier	3.91
**2-hydroxyglutaric acid**	417	137<br>329	21<br>21	Quantifier<br>qualifier	5.7
**itaconic acid**	399	246<br>110	17<br>37	Quantifier<br>qualifier	6.1
**2-hydroxyglutaric acid-$^{13}C_5$**	422	137	21	Quantifier	5.17
**succinic acid-D_4**	391	237.0	17	Quantifier	5.91
**malic acid-$^{13}C_4$**	407	210.0	21	Quantifier	5.63
**fumaric acid-$^{13}C_4$**	389	236.0	15	Quantifier	6.38
**2-oxoglutaric acid-$^{13}C_4$**	554	374.1	25	Quantifier	8.53
**citric acid-D_4**	447	249.0	25	Quantifier	6.28
**pyruvic acid-$^{13}C_3$**	360.0	42.9	25	Quantifier	7.95
**lactic acid-$^{13}C_3$**	227.0	151.8	13	Quantifier	3.91

167
