# Supplementary material for: Pantothenate kinase 4 controls skeletal muscle substrate metabolism
Source: Nat Commun. 2025 Jan 2;16:345. doi: 10.1038/s41467-024-55036-w (PMC11695632; doi:10.1038/s41467-024-55036-w)
Supplement: Supplementary file 2 — Description of Additional Supplementary Files [file 41467_2024_55036_MOESM2_ESM.pdf]

Description of Additional Supplementary Files

File Name: Supplementary Data 1
Description: Metabolites detected by nontargeted metabolomics in tibialis anterior (TA) and gastrocnemius muscles from glucose-stimulated male PanK4 WT and PanK4 mKO mice at age 28 weeks (n = 8 for WT and n = 6 for mKO).

File Name: Supplementary Data 2
Description: Genes detected via whole-genome sequencing in gastrocnemius muscles from glucose-stimulated male PanK4 WT and PanK4 mKO mice at age 28 weeks (n = 8 for WT and n = 6 for mKO).

File Name: Supplementary Data 3
Description: Metabolites detected by nontargeted metabolomics in tibialis anterior (TA) from fasted or fed male C57BL6/J mice overexpressing PanK4 in one TA and MCS (control) in contralateral TA (n = 6).

File Name: Supplementary Data 4
Description: Proteins detected by mass spectrometry in pulldowns targeting endogenous or flag-tagged PanK4 in tibialis anterior (TA) muscle either overexpressing PanK4 or lacking PanK4.
